# Knowledge and associated factors with respect to prevention of post-traumatic compartment syndrome among surgical unit nurses; a multi-center cross-sectional study

**DOI:** 10.1186/s12912-024-01806-2

**Published:** 2024-03-06

**Authors:** Astewil Moges Bazezew, Yalemwork Getahun, Tiruye Azene Demlie, Desalegn Getachew Ayele, Tsehayu Melak Siyoum, Gezahagn Demsu Gedefaw, Kasaye Ahmed Zeleke, Esayas Alemshet Tekletsadik, Sintayehu Simie Tsega, Melkamu Tilahun Dessie, Ashenafi Fekad Getahun, Ashenafi Worku Woretaw

**Affiliations:** 1 Department of Surgical Nursing, School of Nursing, College of Medicine and Health Sciences, University of Gondar, Gondar, Ethiopia; 2 Department of Neonatal Nursing, School of Nursing, College of Medicine and Health Sciences, University of Gondar, Gondar, Ethiopia; 3 Department of Medical Nursing, School of Nursing, College of Medicine and Health Sciences, University of Gondar, Gondar, Ethiopia; 4 Department of Pediatrics and Child Health Nursing, School of Nursing, College of Medicine and Health Sciences, University of Gondar, Gondar, Ethiopia

**Keywords:** Knowledge and post-traumatic compartment syndrome, Nurses, Ethiopia

## Abstract

**Background:**

Delayed recognition of compartment syndrome can result in devastating consequences such as the need for amputation or even death. Nurses are at the frontline of patient care and they must have a high index of suspicion for compartment syndrome. Even though nurses’ knowledge about the prevention of compartment syndrome is important, there are no studies in Ethiopia. Therefore this study aims to assess the knowledge and associated factors of nurses towards the prevention of post-traumatic compartment syndrome.

**Method:**

An institutional-based cross-sectional study was conducted among 410 nurses from 26 April to 25 May 2023 at five Comprehensive Specialized Hospital. A stratified sampling technique was employed to recruit the required participants for the study. The data were collected using a structured self-administered questionnaire. The descriptive statistics were presented in text and tables. Analytical analysis schemes including bivariable and multivariable logistic regression were computed considering P-value < 0.05 to identify statistically significant factors.

**Result:**

Nearly three- fifths (61.6%; 95% CI: 56.7 to 66.3) of nurses had adequate knowledge and significantly associated with being male (AOR: 1.615, 95% CI: 1.050–2.485), nurse use of guidelines (AOR: 2.079, 95% CI: 1.307–3.307), nurses they have been trained (AOR = 1.650; 95 CI: 1.063–2.562), and nurses’ who had more than 15 years’ (AOR: 4.207, 95 CI: 1.762–10.045) experience had good knowledge with respect to prevention of compartment syndrome than the counterparts.

**Conclusion:**

Even though nurses’ knowledge regarding the prevention of post-traumatic compartment syndrome was found to be good, Diligent nursing assessment and monitoring of clinical signs should be critically performed. So, it is better to strengthen training, equip wards with standardized guidelines, and create a safe working environment should be routine activities.

## Introduction

Acute compartment syndrome (ACS) is a surgical emergency defined by a critical increase in pressure within a closed osteofascial compartment [1, 2].The critical level is the intramuscular tissue pressure, which causes the capillary bed to collapse, preventing blood flow through the capillaries into venous drainage [2–4]. This results in compromised tissue perfusion, ischemia, and necrosis. It is predominantly a clinical diagnosis based on the recognition of pain that is out of proportion to the initial injury [5].

The ACS is potentially life threatening and limb-threatening, associated with a mortality rate of 15% and serious morbidity [6]. A systematic review study showed the rate of compartment syndrome in twenty articles ranged from 0 to 30.7% [7]. Delayed recognition of compartment syndrome can result in devastating consequences, such as the need for amputation or even death. Nurses are at the frontline of patient care in the orthopedic department, and they must have a high index of suspicion for compartment syndrome [3, 5].

A critical factor in a poor outcome following ACS is a delay in the initial recognition and diagnosis of the syndrome. One of the main causes of a delay in diagnosis is due to insufficient awareness of this condition from a nursing assessment [3–5]. Therefore, it is necessary for critical care nurses to understand the factors that predispose patients to ACS and to be vigilant when assessing patients to predict and manage patients’ needs appropriately. Even if nurses can play an important role in the early detection of patients at risk of ACS available literature revealed that nurses had significant knowledge gaps [8]. A study carried out in Nepal found that 39 people (43.33%) had a sufficient level of knowledge. On the other hand, a descriptive study design showed that 27% of nurses had good knowledge about cast complications [9]. A nurse must understand the etiologies, high-risk situations, and the immediacy of intervention and have a responsibility to provide early identification and intervention to patients with compartment syndrome in order to allow for the best possible outcomes and to increase patient satisfaction [10].

To facilitate more accurate detection and diagnosis of ACS, the modern intensive care unit has undergone significant growth. A solid understanding of pathophysiology together with knowledge of evidence-based clinical practice guidelines must serve as the foundation for nurses’ knowledge of ACS. It enables them to be aware of common areas, early indications and symptoms, therapies, and escalation strategies, this will improve their baseline knowledge and would make it possible to diagnose the illness as effectively as possible, thereby enhancing patient care [3–5, 8, 11]. Without this fundamental knowledge and understanding, presenting symptoms can be overlooked or misinterpreted as signs of other critical illnesses [12, 13]. It is well known that nursing care is very important, so any little error by nurses while providing care and treatment might cause the patients to pass away. Previous studies showed that qualified nurses who are more knowledgeable about preventing post-traumatic compartment syndrome can improve patient outcomes.

This study was perhaps the first of its kind in Ethiopia and focused on determining the knowledge of nurses working in Northwest Amhara Regional State Referral Hospitals and investigating the factors associated with knowledge. This prevents nurses from fulfilling their responsibility and does not properly remind them of their scope for the job.

Therefore, the current study shows that addressing the identified factors could be helpful in developing suitable strategies to improve nurses’ knowledge regarding post-traumatic compartment syndrome prevention.

The findings may have important policy implications for developing effective strategies to improve nurses’ knowledge of delivering high-quality care. The findings are also provide relevant to professional institutions, which should concentrate on modifying nursing curricula and offering seminars and trainings for professional development to enhance nurses’ understanding of preventing post-traumatic compartment syndrome. This study could also baseline information to next researchers on the knowledge of nurses on the prevention of post-traumatic compartment syndrome and its predictors.

Nurses’ knowledge is influenced by a variety of institutional and socioeconomic factors, including age, marital status, level of education, work experience, guidelines, training, reading updated evidences, and job satisfaction. Thus, the purpose of this study was to assess nurses’ knowledge of the prevention of post-traumatic compartment syndrome at Northwest Amhara Regional State Referral Hospitals in Northwest Ethiopia and explore the factors that may have contributed to that knowledge.

## Methods and materials

### Study design and period

An institutional-based cross-sectional study was conducted in the surgical units of Northwest Amhara comprehensive specialized hospitals from April 26 to May 25, 2023.

### Study area

The study was carried out in Northwest Ethiopia at the comprehensive specialized hospitals of Northwest Amhara Regional State. Northwest Amhara is located in Ethiopia’s northwest region. Tibebegion Comprehensive Specialized Hospital (TGCSH), University of Gondar Comprehensive Specialized Referral Hospital (UoGCSRH), Felegehiwot Referral Hospital (FHRH), Debre Markos Comprehensive Specialized Hospital (DMCSH), and Debre Tabor Comprehensive Hospital (DTCSH) are the five government referral hospitals. Over 22,000,000 residents of the hospitals’ catchment regions can receive both inpatient and outpatient care from them. A total of 745 nurses are employed in emergency departments, including trauma units, operating rooms, recovery wards, surgical wards (including oncology), orthopedic wards, and surgical intensive care units. Out of the 1682 nurses that are now employed by those facilities [14].

### Sources of population and study population

- Nurses who had been working in the Northwest Amhara comprehensive specialized hospitals in Ethiopia.

### Study population

- All nurses who had been working in selected units or wards at UoGCSRH, DTRH, DMRH, TGRH, and FHRH during the data collection period in 2023.

### Eligibility criteria

#### Inclusion criteria

- All nurses who are working at Northwest Amhara comprehensive specialized hospitals during the time of data collection.

### Sample size determination and sampling technique

#### Sample size determination

The sample size of the study is calculated using the formula for the estimation of a single proportion of the population, and the assumption is that the proportion of knowledge of nurses regarding the prevention of post-traumatic compartment syndrome is 50% (since there has been no study conducted in our country), with a 95% level of confidence and a 5% margin of error. By using a z value of 1.96 at 95% CI, the minimum sample size for the study will be:

$$ n = \frac{{{{\left( {Z\alpha /2} \right)}^2} * \left( p \right)\left( {1 - p} \right)}}{{{d^2}}}$$

n = sample size.

p = proportion of knowledge and practice of nurses regarding preoperative patient teaching = 0.5.

d = maximum allowable error (margin of error) = 0.05.

Z = the value the standard normal distribution at the 95% confidence level (z = 1.96).

n = (1.96) (1.96) *(0.5) (1-0.5)/ (0.05) (0.05) = 384 subjects;

None response: 38.4 ≈ 39 (10%). The final sample size was 422.

### Sampling technique

To recruit the required participants for the study, a stratified sampling technique was employed. First, the study participants are stratified by hospital and working ward/unit. After allocated the required sample for each stratum proportionally, a proportional number of participants were selected by a simple random sampling method. All hospital administration and human resources records state that there were 745 nurses employed overall in this ward or unit. Of the 745 samples, 423 were allocated proportionally to the number of nurses employed in each institution. Finally, a simple random technique was used to choose those individuals.

### Operational definition

#### Compartment syndrome

acute compartment syndrome is a surgical emergency characterized by elevated pressure in an unyielding osteofascial compartment [15].

#### Good knowledge

Participants in the study were considered to have high knowledge if their answers to the knowledge questions were either above or equal to the computed median [11].

#### Poor knowledge

Participants in the study were considered to have high knowledge if their answers to the knowledge questions were below to the computed median [11].

### Data collection tool and procedure

A structured, self-administered questionnaire was used to collect data. 22 questions for knowledge on the prevention of compartment syndrome were adopted from a single piece of literature [16]. In addition to the outcome variable the tool contains 11 questions for socio-demographic and work-related variable, that were adapted from different literatures [4, 8, 9, 17]. The questionnaires are prepared in the English language based on the study objectives, focusing on the background information on the prevention of post-traumatic compartment syndrome. Five BSc nurses who are working outside of the study area are recruited for data collection, and two MSc-holder nurses are recruited as supervisors. Overall, the data collection process was coordinated and supervised by the principal investigator.

### Data quality assurance

To ensure the quality of data, one-day training was given to data collectors and supervisors regarding the structured questionnaire (on the objective of the study and how to collect the data). A week before starting the actual period of data collection, there was a pretest on 5% of the sample at Woldia Referral Hospital. Regular supervision was done to check the consistency and completeness of the filled-out questionnaires by the supervisors and principal investigator. Face validity was checked by experts. By using SPSS version 20, Cronbach’s alpha was calculated to test the internal consistency of items, and its values were in the acceptable range, which showed 0.767 for knowledge of post-traumatic compartment syndrome prevention. After the actual data collection process, the collected data were cross-checked for the questionnaires’ consistency and completeness.

### Data processing and analyzing

Questionnaires were checked visually and coded, and the data were entered into Epi Info version 7 and exported into SPSS version 20 for analysis. Frequencies, percentages, and medians with IQR were computed to describe the key variables of the study. Binary logistic regression was run to determine significant relations between independent variables and the dependent variable, and all independent variables that were less than 0.2 in the bivariate analysis were entered into multivariable logistic regressions. A *P*-value of < 0.05 was considered significant for all analyses. AOR with a 95% CI and a *p*-value of < 0.05 were used to declare associated factors with the knowledge of nurses on compartment syndrome prevention.

## Result

### Socio-demographic characteristics of the study participants

In this study, 410 nurses responded to the self-administered questionnaires with a response rate of 97%. Among respondents, 228 (56.2%) were males and 230 (56.09%) were married. In this study, the average (49.5%) of the age group was between 31 and 40. Out of 410 nurses, most of the respondents (64.9%) had a bachelor’s degree, and 164 (40%) of the respondents had 11–15 years of work experience **(**Table 1**).**

**Table 1:** Socio-demographic characteristics of the study participants on prevention of post-traumatic compartment syndrome among surgical unit nurses in Northwest Amhara Comprehensive Specialized Referral Hospitals, Northwest Ethiopia, 2023(*N* = 410)

Variables	Category	Frequency	Percent (%)
Sex	Male	228	55.6
	Female	182	44.4
Marital status	Single	173	42.1
	Married	230	56.09
	Divorced	7	1.7
Age	20–30 years	62	15.1
	31–40 years	203	49.5
	41–50 years	103	25.2
	50 years	42	10.2
Educational status	Masters	82	15.1
	BSc degree	266	64.9
	Diploma	62	20
Working experience	≤ 5 years	41	10
	6–10 years	122	29.8
	11–15 years	164	40
	> 15	83	20.2

### Work-related factors in the prevention of post-traumatic compartment syndrome

Nearly half (51.5%) of participants did not take training about the prevention of post-traumatic compartment syndrome. 124 (30.2%) of the respondents reported that they were working at the emergency ward, 211 (51.5%) of the participants were notified that they took training, and 217 (52.92%) reported that their monthly salaries ranged from 7001 to 9000. Of 410 study participants, three-fifths (60%) of participants offered that there are no guidelines in the working department **(**Table 2**).**

**Table 2:** Work-related factors of the study participants on prevention of post-traumatic compartment syndrome among surgical unit nurses in Northwest Amhara Comprehensive Specialized Referral Hospitals, Northwest Ethiopia, 2023(*N* = 410)

Variables	Category	Frequency	Percent (%)
Current Working area	Orthopedic	61	14.9
	Surgical	41	10
	Recovery	102	24.9
	Emergency	124	30.2
	Others	82	20
Working place	DTCSH	39	9.5
	DMCSH	65	15.85
	TGCSH	86	20.97
	UoGCSH	92	22.43
	FHCSH	128	31.2
Daily working hours	≤ 8 h	259	63.8
	≥ 9 h	147	36.2
Training	Yes	221	53.9
	No	189	46.1
Guideline/protocol	Yes	164	40
	No	246	60
Monthly salary	≤ 5000	33	8.1
	5001–7000	104	25.36
	7001–9000	217	52.92
	≥ 9000	58	14.14

### Nurses’ knowledge regarding the prevention of post-traumatic compartment syndrome

The overall median knowledge score of the study participants on prevention of post-traumatic compartment syndrome was 11. In this study, 250 (61.0%), with a 95% CI (56.23–65.72) of the participants, had good knowledge. Among a total of knowledge assessment questions, the majority (84.9%) of participants correctly answered the statement that compartment syndrome is commonly seen in tibia fractures, and three-fourths (75.4%) of the participants correctly answered what traumatic limb compartment syndrome is 289 (70.5%) of the participants gave the correct answer that the possible sites for developing compartment syndrome are the lower leg, forearm, and wrist. Only 102 (24.9%) correctly answered the statement that after application of the cast in the upper extremities, how should the nurse position the client’s limb for the first 24 h to prevent compartment syndrome. **(**Table 3**)**

**Table 3:** Nurse’s responses on knowledge of prevention of post traumatic compartment syndrome in Northwest Amhara Comprehensive Specialized Referral Hospitals, Northwest Ethiopia 2023 (*N* = 410)

Statements on knowledge of post traumatic compartment syndrome	Correct		Incorrect	
	N	%	N	%
What is traumatic limb compartment syndrome?	309	75.4	101	24.6
Compartment syndrome is commonly seen on	348	84.9	62	15.1
which of the following are common site for compartment	226	55.1	184	44.9
which of the following are possible site for developing compartment syndrome	289	70.5	121	29.5
which of the following is the main conditions that cause compartment syndrome	244	59.5	166	40.5
Which of the following characteristics of the fascia can because it develop compartment syndrome?	145	35.4	265	64.6
what should you recommend for a patient who has a compartment syndrome	207	50.5	203	49.5
what is the first sign of compartment syndrome	288	70.2	122	29.8
which of the following is the late sign of compartment syndrome	205	50	205	50
In compartment syndrome, with comprised blood supply creating ischemia, irreversible muscle damage occurs within……………	227	55.4	183	44.6
Which of the following nursing consideration is used to assess the compartment syndrome	164	40	246	60
Which of the following is the diagnostic criterion of the compartment syndrome?	227	55.4	183	44.6
One of the common complications of compartment syndrome is	247	60.2	163	39.8
A surgical procedure done to relieve pressure in compartment syndrome is	163	39.8	247	60.2
Statements on knowledge on the prevention of post traumatic compartment syndrome				
When assessing the client’s fractured extremity, if the nurse is unable to assess the capillary refill in the beds what should the nurse do?	103	25.1	307	74.9
Which of the following 6 P’s are associated with compartment syndrome?	146	35.6	264	64.4
Which of the following is the main nursing consideration to prevent the development of compartment syndrome?	124	30.2	286	69.8
If the patient is on skin traction, what nursing action should be to prevent compartment syndrome	123	30	287	70
After application of cast in upper extremities, how should the nurse position the client’s limb for the first 24 h to prevent compartment syndrome?	102	24.9	308	75.1
A client who has had a plaster of Paris cast applied to his forearm is receiving pain medication. To detect early manifestations of compartment syndrome, which of the reassessments should the nurse make?	104	25.4	306	74.6
While caring for a client with a newly applied plaster of Paris cast, the nurse makes note of all the following conditions. Which assessment finding requires immediate notification of the physician	145	35.4	265	64.6
Which one of the following is the method of assessing for the sign of circulatory impairment in a client with a fractured femur is to ask the client to?	267	65.1	143	35.9

### Factors associated with the level of knowledge of prevention of post-traumatic compartment syndrome

Five of the eleven variables in the binary logistic regression were found to be significantly associated with participants’ knowledge about preventing post-traumatic compartment syndrome, with a *p*-value of less than 0.2. However after adjusting for the effects of potentially confounding variables using multivariate logistic regression, it was found that nurses’ knowledge of preventing post-traumatic compartment syndrome was significantly predicted by their training, adherence to guidelines, and work experience.

In the present study, being male (AOR: 1.615, 95% CI: 1.063–2.562), having training of the prevention of acute compartment syndrome (AOR = 1.650; 95 CI: 1.063–2.562), nurses adhering guidelines (AOR: 2.079, 95% CI: 1.307–3.307), and having working experience ≥ 15 years (AOR: 4.207, 95 CI: 1.762–10.045) were significantly associated with their knowledge. **(**Table 4**)**

**Table 4:** Variable and multivariable analysis of factors associated with knowledge of nurses on prevention of post traumatic compartment syndrome in Northwest Amhara Comprehensive Specialized Hospitals, Northwest Ethiopia 2023 (410)

Variables		Knowledge of nurses		COR(95%CI)	AOR(95%CI)	*p*-value
		Good	Poor			
Sex	Female	81	101			
	Male	149	79	1.513(1.014–2.256)	1.615(1.050–2.485)	0.029**
Working experience	≤ 5 years	20	21	1		
	6–10 years	62	60	1.085(0.535-2.202)	1.111(0.518–2.381)	0.787
	11–15 years	101	63	1.683(0.846-3.351)	1.697(0.809–3.558)	0.162
	> 15 years	67	16	4.397(1.936–9.983)	4.207(1.762–10.045)	0.001**
Monthly salary	≤ 5000	18	15	1		
	5001–7000	51	53	0.777(0.347-1.739)	0.458(0.176-1.188)	0.108
	7001–9000	134	83	1.346(0.630-2.874)	0.953(0.379-2.393)	0.918
	≥ 9000	49	8	1.765(1.994–16.68)	2.906(0.839-10.059)	**0.092**
Having guidelines	No	131	115	1		
	Yes	149	15	2.321(1.518–3.550)	2.079(1.307–3.307)	0.002**
Took training	No	97	92	1		
	Yes	153	68	2.134(1.425–3.195)	1.650(1.063–2.562)	0.026**

Variables show that significant association during multivariable logistic regression at**

*P*-value < 0.05 1 = reference

## Discussion

It is important to improve the nurse staff’s knowledge of acute compartment syndrome to prevent it [18]. The result of this study showed that good knowledge of nurses on the prevention of post-traumatic compartment syndrome was 250 (61.0%) with a 95% CI of 56.23–65.72. This study is lower than the study conducted at Kom Hamada and Itay El Baroad Hospital, Egypt, 81.3% [17]. Since a quasi-experimental research methodology was employed, this is probably the cause of the discrepancy. Because to elucidate, refresh, and master nurses’ knowledge, teaching is essential. Different literature states that continuous educational programs should be planned for nurses to enhance their knowledge and achieve a high quality of care [13]. Yet, this result is more than that of a study carried out at Helwan University in Egypt, where 80% of the study nurses possessed inadequate knowledge [13]. This finding is also more than that of a study carried out at Patan Hospital, which found that 39 people (43.33%) had a sufficient level of understanding [16]. The difference for this reason might be the application of different analysis model which is, in the previous study ordinal logistic regression was employed and Out of 90 nurses, 38 (42.22%) had an adequate level of knowledge, 29(32.22%) had a moderate level of knowledge and 23(25.56%) had an inadequate level of knowledge, while in our study binary logistic regression was employed.

Regarding the determinants of the level of knowledge on the prevention of post-traumatic compartment syndrome, this study found that male nurses were found to have good knowledge of prevention of post-traumatic compartment syndrome by 1.615 times as compared to females. One possible explanation for this could be that women often do more household chores, including cooking, cleaning, and child care, as well as carrying greater weight. So, they might not have enough time to increase their knowledge because of their workload from other extracurricular activities at home.

Those nurses who received training related to the prevention of compartment syndrome were 1.650 times more likely to have good knowledge on the prevention of post-traumatic compartment syndrome as compared to their counterparts. This is supported by the previous study [8], it emphasizes the critical importance of continuous training and education for nurses in order to increase awareness and facilitate early identification of the problem. This is because the nurse’s knowledge and practice were improved and affected by the training program [19]. The possible reason might be that training plays an important role in improving the quality of patient care. Promoting the efficacy of nurses’ on- and off-site training is an essential requirement since it is necessary to update theoretical and practical knowledge in every aspect of the nursing profession. In this study, nurses who use guidelines had 2.059 times better knowledge than those who do not use them. This might mean that clinical guidelines are evidence-informed recommendations intended to enhance patient care, and a valid guideline has the potential to influence care outcomes [20].

They make caregivers aware of interventions that lack solid evidence to support them, emphasize the value and techniques of critical evaluation, and focus attention on harmful, inefficient, and inefficient procedures. Clinical suggestions are useful in supporting quality-improving activities. In a similar vein, these guidelines enable patients to choose the best course of action based on their requirements and preferences and to make more educated healthcare decisions [21].

The other factor that influences the prevention of post-traumatic compartment syndrome is working experiences. In these findings nurses who have worked for more than 15 years have 4.207 times a higher level of knowledge of the prevention of post-traumatic compartments than those nurses who have ≤ 5 years of working experiences. It is true that some literature findings showed that more experienced nurses were more likely knowledgeable. This may be because health professionals are exposed to more situations as their years of practice grow, and they get more experience by working with senior staff members [22]. Another study states that respondents with work experience of ≥ 10 years were 2.7 times more likely to have good knowledge as compared to nurses who had less than 10 years of work experience (81.34). This might be because experience increases the chance for trainees to get up-to-date information about patient care [23]. However, according to a study on the impact of work experience on professional nurses, those with more than 20 years of experience placed a lower emphasis on professional values [24]. This could be due to several factors, such as the length of time since their official training. Despite nurses’ strong desire for ongoing education, this education may be more focused on meeting their needs in the workplace.

### Strength and limitation

The limitation of this study is that it may not show a temporal relationship because of the cross-sectional study design. Despite the limitation, this study covers a large setting (multicenter) area, which helps with generalization is more representative of the finding, which is basically nurses who are working in a study setting, which is Northwest Amhara Regional State Referral Hospitals, Northwest Ethiopia and clearly showed the knowledge and associated factors of post-traumatic compartment syndrome prevention among nurses working at comprehensive referral hospitals for the first time in Ethiopia. It will be an essential source of information for policymakers as they create appropriate policies and provide a baseline of knowledge on nurses’ awareness of post-traumatic compartment syndrome prevention.

## Conclusion

This study revealed that nurses’ knowledge regarding the prevention of post-traumatic compartment syndrome was acceptable in comparison with the available literature. A positive and substantial association was found between having an adequate understanding of preventing post-traumatic compartment syndrome and being male, adhering to guidelines, attending training, and working as a nurse for at least 15 years.

## Data Availability

No datasets were generated or analysed during the current study.

## Abbreviations

ACS: Acute Compartment Syndrome
AOR: Adjusted Odds Ratio
BSc: Bachelor of Science
CI: Confidence Interval
COR: Crude Odds Ratio
DMRH: Debre Markos Hospital
DTRH: Debre Tabor Referral Hospital
Epi- Info: Epidemiological Information
FHRH: Felege Hiwot Referal Hospital
SPSS: Statistical Package Software for Social Sciences
TGRH: Tibebe Gion Referral Hospital
UoGCSRH: University of Gondar Comprehensive Specialized Hospital

## References

[1] Dowd S. The Critical Role of Nursing Assessment in Clinical Outcomes of Acute Compartment Syndrome: An Integrative Literature Review. 2019.

[2] Limbert E, Santy-Tomlinson J (2017). Acute limb compartment syndrome in the lower leg following trauma: assessment in the intensive care unit. Nurs Standard.

[3] Khajuria A, Shah R, Gbejuade H, Siddiqui S. Increasing awareness of compartment syndrome among orthopedic nurses and trauma nurse practitioners at a district general hospital: a complete audit loop. Clin Audit. 2017;9(9).

[4] Pokharel K (2020). Knowledge regarding prevention of post traumatic compartment syndrome of limbs among nurses of a teaching hospital. J Patan Acad Health Sci.

[5] Robertson C, Baggott J, Duncan J. A quality improvement project to assess and improve the recognition of compartment syndrome by nurses in the orthopedic department. Cureus. 2020;12(10). 10.7759/cureus.11179 PMC7689952 33262915

[6] Orlin JR, Lied IH, Stranden E, Irgens HU, Andersen JR (2016). Prevalence of chronic compartment syndrome of the legs: implications for clinical diagnostic criteria and therapy. Scandinavian J pain.

[7] Heemskerk J, Kitslaar P (2003). Acute compartment syndrome of the lower leg: retrospective study on prevalence, technique, and outcome of fasciotomies. World J Surg.

[8] Khajuria A, Shah R, Gbejuade H, Siddiqui S. Increasing awareness of compartment syndrome among orthopedic nurses and trauma nurse practitioners at a district general hospital: a complete audit loop. Clin Audit. 2017:9–17.

[9] Ahmed A, Hussein H (2016). Nurses knowledge toward cast complications in orthopedic ward at Al-Najaf AL-Ashraf hospitals. Int J Sci Res Publ.

[10] Garner MR, Taylor SA, Gausden E, Lyden JP (2014). Compartment syndrome: diagnosis, management, and unique concerns in the twenty-first century. HSS Journal®.

[11] Walls MH (2017). Compartment syndrome: an orthopedic emergency. J Emerg Nurs.

[12] Pechar J, Lyons MM (2016). Acute compartment syndrome of the lower leg: a review. J Nurse Practitioners.

[13] Reyad OR, Mahmoud FH, Eldriny SNM (2022). Assessment of nurses’ knowledge and practice regarding intra-abdominal pressure measurement and Abdominal Compartment Syndrome Prevention. Egypt J Hosp Med.

[14] Bayleyegn B, Mehari A, Damtie D, Negash M. Knowledge, attitude and practice on hospital-acquired infection prevention and associated factors among healthcare workers at university of gondar comprehensive specialized hospital, northwest Ethiopia. Infection and drug resistance. 2021:259– 66. 10.2147/IDR.S290992 PMC7850400 33536767

[15] Pearse MF, Harry L, Nanchahal J. Acute compartment syndrome of the leg: fasciotomies must be performed early, but good surgical technique is important. British Medical Journal Publishing Group; 2002. pp. 557–8. 10.1136/bmj.325.7364.557 PMC1124092 12228118

[16] Pokharel K, Knowledge regarding post traumatic compartment syndrome, of limbs among nurses of teaching hospital (2020). Emergency.

[17] Mahran GSK, Abozied SA-S, Ahmed GH, Abd El-Hakeem EE, Abd El-Hafez AI. Effect of Teaching Program on nurses’ knowledge and skills and development of Abdominal Compartment Syndrome among Intensive Care patients. e-ISSN. IOSR Journal of Nursing and Health Science (IOSR-JNHS); 2018. pp. 2320–1959.

[18] Boesgaard-Kjer DH, Boesgaard‐Kjer D, Kjer JJ (2013). Well‐leg compartment syndrome after gynecological laparoscopic surgery. Acta Obstet Gynecol Scand.

[19] Abdelazeem E, Fashafsheh I, Fadllalah H (2019). Effect of training program on nurses knowledge and competence regarding endotracheal tube and tracheostomy care in mechanically ventilated patients. Int J Nurs.

[20] Brouwers MC, Florez ID, McNair SA, Vella ET, Yao X, editors. Clinical practice guidelines: tools to support high quality patient care. Seminars in nuclear medicine. Elsevier; 2019. 10.1053/j.semnuclmed.2018.11.001 30819394

[21] Woolf SH, Grol R, Hutchinson A, Eccles M, Grimshaw J (1999). Potential benefits, limitations, and harms of clinical guidelines. BMJ.

[22] Desta M, Ayenew T, Sitotaw N, Tegegne N, Dires M, Getie M (2018). Knowledge, practice and associated factors of infection prevention among healthcare workers in Debre Markos referral hospital, Northwest Ethiopia. BMC Health Serv Res.

[23] Biresaw H, Asfaw N, Zewdu F (2020). Knowledge and attitude of nurses towards patient safety and its associated factors. Int J Afr Nurs Sci.

[24] Fernández-Feito A, Palmeiro-Longo MR, Hoyuelos SB, García-Díaz V (2019). How work setting and job experience affect professional nurses’ values. Nurs Ethics.

